# Experimental Evaluation of Shark Detection Rates by Aerial Observers

**DOI:** 10.1371/journal.pone.0083456

**Published:** 2014-02-03

**Authors:** William D. Robbins, Victor M. Peddemors, Steven J. Kennelly, Matthew C. Ives

**Affiliations:** 1 Cronulla Fisheries Research Centre of Excellence, New South Wales Department of Primary Industries, Cronulla, New South Wales, Australia; 2 Wildlife Marine, Perth, Western Australia, Australia; University of Muenster, Germany

## Abstract

Aerial surveys are a recognised technique to identify the presence and abundance of marine animals. However, the capability of aerial observers to reliably sight coastal sharks has not been previously assessed, nor have differences in sighting rates between aircraft types been examined. In this study we investigated the ability of observers in fixed-wing and helicopter aircraft to sight 2.5 m artificial shark analogues placed at known depths and positions. Initial tests revealed that the shark analogues could only be detected at shallow depths, averaging only 2.5 m and 2.7 m below the water surface for observers in fixed-wing and helicopter aircraft, respectively. We then deployed analogues at shallower depths along a 5 km-long grid, and assessed their sightability to aircraft observers through a series of transects flown within 500 m. Analogues were seen infrequently from all distances, with overall sighting rates of only 12.5% and 17.1% for fixed-wing and helicopter observers, respectively. Although helicopter observers had consistently higher success rates of sighting analogues within 250 m of their flight path, neither aircraft observers sighted more than 9% of analogues deployed over 300 m from their flight paths. Modelling of sighting rates against environmental and experimental variables indicated that observations were affected by distance, aircraft type, sun glare and sea conditions, while the range of water turbidities observed had no effect. We conclude that aerial observers have limited ability to detect the presence of submerged animals such as sharks, particularly when the sharks are deeper than ∼2.6 m, or over 300 m distant from the aircraft's flight path, especially during sunny or windy days. The low rates of detections found in this study cast serious doubts on the use of aerial beach patrols as an effective early-warning system to prevent shark attacks.

## Introduction

Aerial surveys using helicopter and fixed-wing aircraft have been used to estimate the presence and abundance of terrestrial and marine animals for many years. Terrestrial surveys have focused on large quadrupeds such as moose, oryx, elk, deer, horses and zebras [1]–[6], although abundances of smaller animals such as kangaroos, goats, emus and smaller birds have also been assessed [7]–[11]. Numerous factors affect the ability of observers to sight terrestrial species, including group size, individual activity and the frequency at which animals are obscured by vegetation [4], [12], [13].

Although not affected by many of the factors involved in terrestrial aerial surveys, aerial sighting rates of marine animals are influenced by their own suite of environmental and biological factors. Water turbidity, wind strength and sea chop can all reduce sighting rates [14], [15], as can the size and behaviour of the animals. Marine aerial surveys have generally focused on the abundance of air-breathing animals, such as bottlenose dolphins, right whales, sea lions, harbour seals, dugongs and turtles [16]–[22]. Sightings of such species are easier than for submerged species like sharks, because they spend at least some time on the surface [14]. Sighting animals as they surface to breathe reduces the obscuring effects of turbidity, and creates additional sighting cues such as a high-contrast wake as individuals break the surface. This effect is enhanced when surveying species such as dolphins travelling in pods, where thousands of individuals may be present in a single group [23].

Targeted aerial surveys of sharks have focused mostly on very large (≥10 m) species, such as whale sharks (*Rhincodon typus*) and basking sharks (*Cetorhinus maximus*) [24]–[27]. These species frequent the surface for feeding and courtship [28], [29], allowing groups of individuals to be readily detected. However, most shark species are much smaller, generally do not form aggregations, and spend much of their time below the surface of the water [30], [31]. This makes them a difficult target for aerial observers to detect and identify. While smaller shark species such as blue sharks (*Prionace glauca*) and hammerhead sharks (*Sphyrna* spp) have been recorded in published aerial surveys [32], sharks are generally absent or are reported in low numbers in aerial marine surveys [23].

Aerial shark detection for public safety occurs in Australia using both helicopters and fixed-wing aircraft. Although these aerial beach patrols are not formal surveys for quantifying the abundances of sharks, their role as a means for protecting swimmers from attack means they should ideally detect a high proportion of sharks present in the area overflown. Australian aerial patrols survey large expanses of beach, receive considerable public support as a perceived form of protection against shark attack and resulting shark sightings often receive considerable media attention. However, because potentially dangerous coastal sharks such as white sharks and tiger sharks may spend much of their time close to the substratum [30], [33], the reported sightings from aerial surveillance may represent only a small proportion of the sharks actually present.

As part of a process to review the suitability of aerial beach patrols in NSW we undertook a structured assessment of shark sighting rates by observers in both helicopters and fixed-wing aircraft, using comparable conditions (speed, altitude and cockpit configuration) to those employed during aerial beach patrols. As the real-time tracking of live sharks was logistically impractical, we assessed aerial sighting effectiveness using life-sized plywood shark analogues. Artificial animal analogues have been successfully used to calibrate previous marine surveys [15] and allowed us to control the depth and spatial distribution of potential sightings while providing a realistic visual image for aircrew observers. We initially assessed the depths at which the shark analogues were sighted by fixed-wing and helicopter observers and, using this information, investigated the effects of aircraft distance and environmental variability on sighting rates.

## Materials and Methods

### Ethics statement

All fieldwork was conducted with permission from the NSW Marine Parks Authority and the Royal Australian Navy. Animal ethics were not required for this study.

### Location and equipment

The study was carried out at the northern side of Jervis Bay, NSW (35.0167°S, 150.7311°E). This is a large embayment (∼112 km^2^), known for its relatively clear inshore waters, with a substratum consisting primarily of white sand and seagrass and a topography offering protection from winds and swells.

A Cessna 182 fixed-wing aeroplane and a Robinson R44 Clipper II helicopter were used in this study. The fixed-wing company had considerable experience with aerial shark detection, while the helicopter company had no prior experience. The aircrews of both aircraft consisted of a pilot, an observer to sight the shark analogues and a data recorder. The observers all wore polarised sunglasses to aid analogue sightings. Due to cockpit configurations, the observer looked out to the right in the fixed-wing aircraft and to the left in the helicopter. Both aircraft were flown at a constant height of 500 ft (∼152 m), to replicate the altitude of standard aerial beach patrols.

The artificial shark analogues consisted of 2.5 m (total length) plywood cutouts, painted a similar shade of grey to that of large sharks. The analogue shape was traced from a white shark (*Carcharodon carcharias*) incidentally captured through the NSW Department of Primary Industries Shark Meshing (Bather Protection) Program. The head morphology was altered in a number of analogues to mimic blunt-headed tiger sharks (*Galeocerdo cuvier*) and hammerhead sharks (*Sphyrna* sp). The hammerhead outline was traced from a stored head, while the tiger shark head outline was drawn from a photograph. Wire cables of sufficient length to allow the analogues to sit approximately horizontal in the water column were attached to each corner, terminating in a single metal ring to which an anchor rope was attached. The analogues were inherently buoyant, although a small (3 cm) surface float was attached via a string to aid the boat crew in relocating submerged analogues. These floats were not visible to the aircraft observers.

### Depth experiment

Depth trials were carried out between 0800 hr and 1600 hr over two days, with an equal proportion of sunny and overcast conditions, and wind strengths under 10 kts each day.

A series of stations consisting of a small galvanised pulley attached to 28 kg of 40 mm-gauge anchor chain as an anchor were deployed in 6 m and 12 m water depths. A 6 mm rope was threaded through the pulley, with one end attached to a shark analogue, and the other end held taut by a crewmember on a 5.7 m runabout vessel anchored ∼20 m away. Each analogue was initially sunk to a depth of 5–6 m, after which an aircraft would then orbit (fixed-wing) or hover (helicopter) at 500 ft (∼150 m) above the position of the analogue as it was slowly raised towards the surface by the boat crew member releasing the rope. The aircrew radioed the boat once the analogue was seen, at which time the depth of the analogue below the water surface was digitally measured. The boat crew member randomly changed the speed of the surfacing analogue to prevent it becoming visible to the aircrews after a predictable period.

Each analogue type (white shark, tiger shark and hammerhead shark) was tested six times in each of the two water depths for each aircraft. Water turbidity was estimated using a 25 cm secchi disk deployed in the shade of the boat. Turbidity readings were collected across the day, however for logistical reasons measurements could not be taken in conjunction with analogue testing. The depth at which an analogue was first sighted was modelled using the linear mixed effects regression ‘lme’ function in R [34]. Here, sighting depth was modelled against aircraft type, analogue type, water depth, sun position (calculated on the hour of day), cloud cover and wind speed, with day set as a random factor. The best model was chosen based on AIC values using backward elimination (or top-down) stepwise regression [35], with the complete model being:
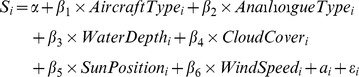



where S_i_ is the dependent variable for the depth at which each analogue was aerially detected and *a*_i_ is the random intercept for days.

### Distance experiment

An east-west 5 km×0.25 km deployment grid was established using a hand-held Garmin GPS. Analogues were anchored at pre-determined positions within the grid, using 14 kg of 40 mm-gauge anchor chain anchor attached to a 6 mm rope. Following the results of the depth trial, all analogues were deployed at 1.8–2.2 m below the water surface, depending on the state of the tide. This ensured that all analogues were potentially visible to aircraft observers.

Six parallel 5 km-long flight transects, 150 m apart were established using GPS. These transects also ran in an east-west orientation to minimise sun glare to the sideways-looking observers. Two transects lay to the north of the deployment grid, two transects lay to the south, and two transects lay within the deployment grid. Any analogues positioned on the non-viewing side of the aircraft were excluded from calculations when examining sighting rates of the innermost two transects.

Three discrete trials were carried out by each aircraft each day. For each trial, the aircraft flew all six transects at 500 ft (∼150 m), in the direction that ensured the observer was always facing towards the centre of the grid. Between three and nine analogues were deployed for each trial, with analogues added/removed by the boat crew between trials once the aircraft left the area. Analogues were placed at least 500 m apart, giving a minimum of ∼10 sec between potential sightings while flying at 100 kts (185 kph). The same analogue configurations were employed for both aircraft each day, although in reverse order. Each day, each aircraft would undertake two trials at their cruising speed (100 kts for fixed-wing, 60 kts [111 kph] for helicopter), without stopping or deviating from their flight path. The third trial allowed the fixed-wing aircrew to orbit to confirm sightings if desired, following the normal procedure during NSW aerial beach patrols. The third helicopter trial was conducted at an increased speed of 100 kts without stopping or deviating from their flight path to allow direct comparison with the fixed-wing aircraft. The order in which the trials were undertaken changed each day, and the order in which each aircraft flew alternated each day. The order in which transects were flown also varied among trials.

When a shark analogue was sighted, the observer would notify the rest of the aircrew, signalling the pilot to mark a GPS waypoint, and the recorder to note both the GPS waypoint number and the observer's best estimate of the angle and distance of the analogue relative to the aircraft. This allowed us to later determine which analogue the observer had sighted. Unless permitted, the aircraft would not deviate from its flight path. Pilots would radio the number of analogues seen at the end of each transect, which was later verified against the datasheets. Datasheets were faxed to the lead author by aircrew at the end of each day, with the latitude and longitude of each GPS waypoint included.

To allow for human error and aircraft yaw during analogue sightings, four positions were calculated for each sighting: 1) the given GPS waypoint; 2) the position at the far side of the deployment grid with a 30° leeway from the angle the observer recorded; 3) the position of the aircraft 2 sec prior to the GPS waypoint (our estimate of the maximum likely delay in the pilot responding and marking the point); and 4) the position at the far side of the deployment grid 2 sec prior to the GPS waypoint, again with a 30° leeway. This produced the coordinates for a four-sided quadrilateral, the boundary of which accounted for any likely errors in marking the position or estimating the angle to the sighted analogue. Using Earth Point online software (https://www.earthpoint.us), the quadrilateral coordinates for each reported sighting were uploaded in Google Earth, along with the positions of deployed analogues for that trial. If an analogue lay within the area bounded by the quadrilateral, it was counted as a validated sighting. If not, it was counted as a false sighting. This procedure was repeated for reduced leeways of 15° and 22.5° to examine the accuracy of the observers' angle estimates. Straight-line distance from the aircraft to each validated analogue sighting was then calculated. Here, half the maximum likely delay (1 sec) was subtracted from each given GPS position to allow for the realistic delay in the pilot reacting, locating and pressing the GPS button following the observer's announcement of a sighting.

The proportion of deployed analogues sighted was calculated manually. To determine the number of potential sightings at each distance, the minimum distance the aircraft passed by the analogue was used if the analogue was not seen, while the straight-line distance from the analogue to the aircraft was used if the analogue was sighted (for example, if the aircraft flew within 100 m of an analogue without it being seen, it was counted as a potential 100 m sighting. However, if the same analogue was sighted from 130 m distance, it was counted as a 130 m sighting). Results were binned into 50 m categories for plotting, with binomial standard errors of sighting rates calculated manually.

Finally, various experimental and environmental variables were modelled against a binary dependent variable defining whether a particular analogue was seen on a particular run. Estimates of wind speed and cloud cover were collected by aircrew and boatcrew when possible for each trial. Water turbidity was measured by the boat crew deploying a secchi disk in the shade of the boat at the start, middle and end of each day's sampling, at both ends and in the middle of the grid. These secchi disk readings were used to estimate the turbidity levels around the analogues at the time the aircraft was passing. To eliminate the tide influencing our depth measurements, water depth for each analogue position was calculated from our GPS points and a high-resolution chart of Jervis Bay. Each of these variables, along with the type of aircraft, the direction the observers were facing (North/South), sighting distance, sun position and day (as a random factor) were used in a binomial generalised linear mixed model to determine which factors contributed to the sighting of each analogue on any particular run (R function lmer, family = binomial) [34]. Once again the best model was chosen based on AIC values using backward elimination stepwise regression. The initial model was:
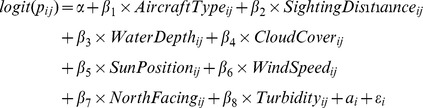



where *p_ij_* is the probability that an analogue was spotted on run *j* of the day *i* and *a*_i_ is the random intercept for days.

The distance trials were conducted between 0940 hr and 1605 hr over six non-consecutive days. Two fixed-wing trials were cancelled due to adverse weather on the first day, with a total of 96 fixed-wing transects (66 transects at 100 kts plus 30 transects at 100 kts with orbiting permitted), and 108 helicopter transects (72 transects at 60 kts plus 36 transects at 100 kts) being flown. There were 230 analogue deployments (107 during fixed-wing trials, 123 during helicopter trials) positioned 0–500 m from the transect paths. These provided 617 potential sighting opportunities for fixed-wing aircraft observers and 709 opportunities for helicopter observers.

## Results

### Depth experiment

**Figure 1:**
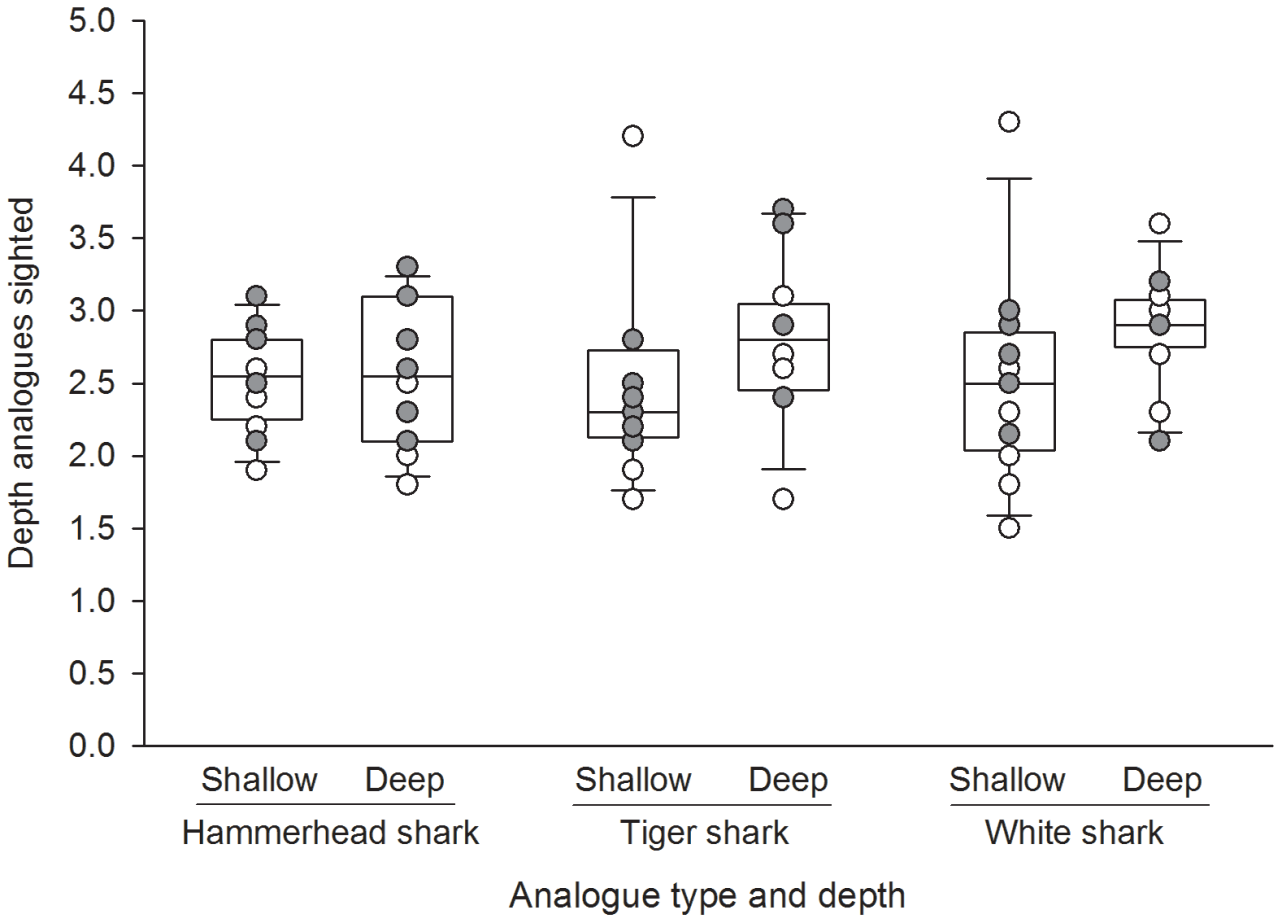
Depths at which shark analogues were sighted by fixed-wing and helicopter observers maintaining their position at 500 ft (∼150 m) around a known position. Overlaid are individual data points from each aircraft; open circles represent fixed-wing sightings, closed circles represent helicopter sightings.

The average depth at which the 2.5 m-long analogues were observed was quite shallow, being 2.5±0.1 m (SE) for the fixed-wing aircraft, and 2.7±0.1 m (SE) for the helicopter. The smaller 25 cm secchi disk turbidity measurements were all greater than these sighting depths (2.9–3.5 m day 1; and 4.5–6.1 m day 2), suggesting that turbidity was not limiting the aircraft observer's sightings of the analogues. The maximum depth of any individual sighting was 4.3 m (fixed-wing) and 3.7 m (helicopter) (Fig. 1).

Based on Akaike Information Criteria (AIC), the most parsimonious model predicting the depth at which analogues were first sighted found water depth to be the only significant variable (Table 1). In this model the sighting depths of analogues increased with water depth, suggesting that the white sands of Jervis Bay did not aid analogue sighting at shallower depths. However, this relationship was very weak, and almost non-significant (Table 1). We conclude that water depth is unlikely to have much influence on the success of analogue sightings; nevertheless we included it as a variable in the subsequent distance trial to examine its effects during standardised aerial patrols.

**Table 1:** Linear regression analysis examining the effects of aircraft type, shark analogue type, water depth, day and various environmental variables on sighting depth.

Coefficients	Estimate	s.e.	t-value	P (>|t|)
(Intercept)	2.74	0.17	15.90	<0.001
Water depth	−0.24	0.12	−1.99	0.050

Note the exact p-value for Water depth is 0.0497.

AIC = 121.6, BIC = 130.6.

The best model was determined based on AIC and likelihood ratio tests with a number of variations in model structure examined including the addition of an interaction between the aircraft type and wind and a random intercept and slope model. An examination of the residuals for the best model showed no signs of serious heteroscadasticty when residuals were compared to fitted values. Normality of the residuals was also considered a reasonable conclusion following an examination of Q-Q plots.

Our model found no significant difference between aircraft types, indicating that observers in each aircraft had comparable ability to sight the shark analogues (Table 1). Changes in the head shapes of analogues also had no significant effect on their sightability (Table 1, Fig. 1). Their equal detection probabilities enabled deployment of all three analogue types in the subsequent distance experiment. Analogues were deployed at shallower depths in the distance experiment (1.8–2.2 m below the water surface) to ensure they could all be potentially sighted.

### Distance experiment

Validated analogue sightings were identified at distances up to 506 m (fixed-wing) and 755 m (helicopter). However, overall sighting rates were low, with only 12.5% and 17.1% of all deployments detected by the fixed-wing and helicopter observers, respectively. Although 15% more analogues were deployed during helicopter trials, their observers recorded 57% more validated analogue sightings than the fixed-wing aircrew. The accuracy of the observers' angle estimates was relatively high for both aircraft, with only a 5% decrease in the number of validated analogue sightings if the leeway was reduced to 22.5°, and further 9% reduction with a 15° leeway. There was no significant difference in sighting rates by either aircraft observers between the smallest and largest leeways (k-s tests; z = 0.426, p = 0.993 for both aircraft types). False sightings (where no analogues lay within the calculated sighting area) accounted for 15% of reported sightings.

**Figure 2:**
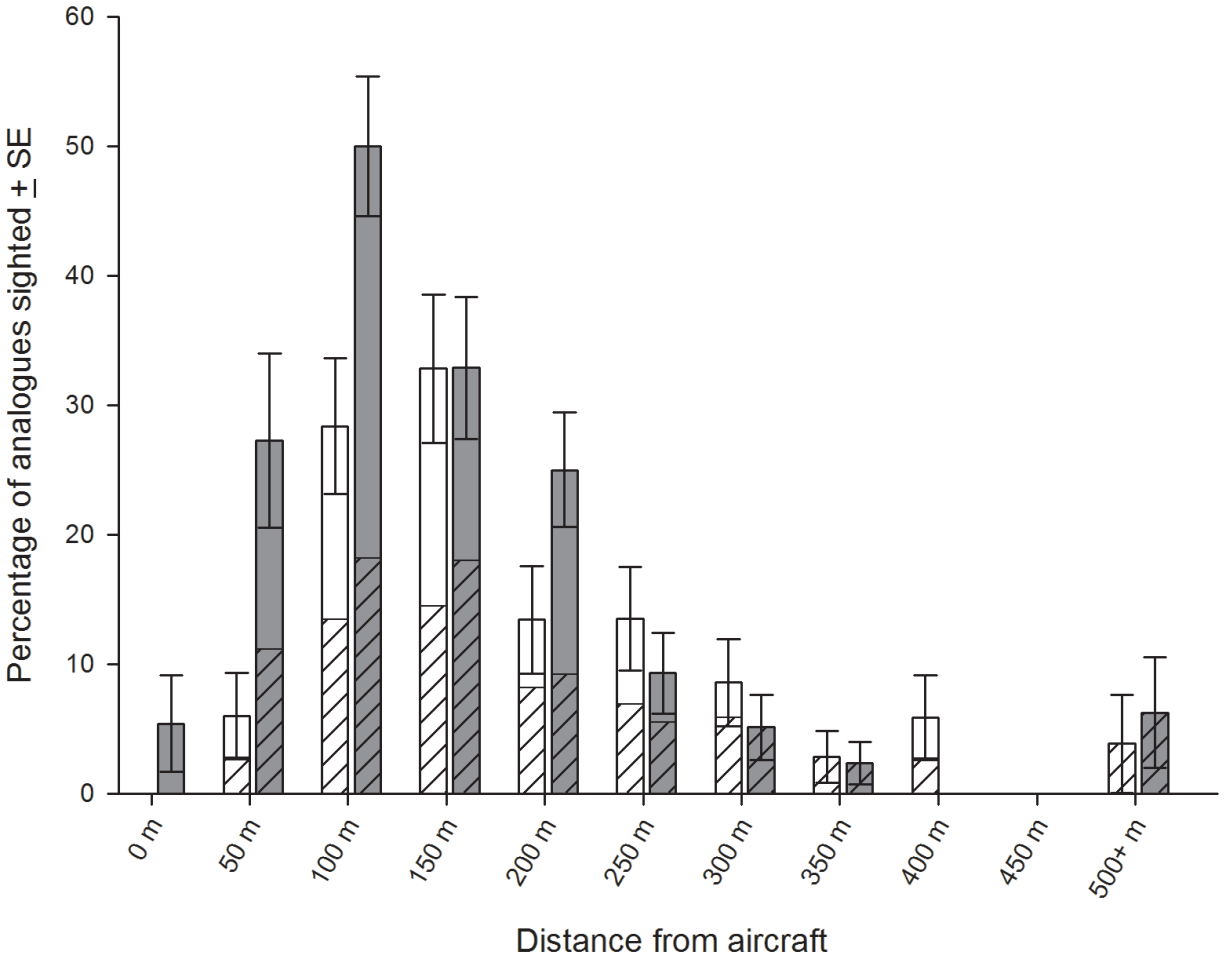
Percentage of validated analogue sightings per aircraft with the relative contribution of different trial treatments. Open bars indicate fixed-wing data, closed bars indicate helicopter sightings. Hashed bars indicates contribution of trials using standard methodology (cruising speed, no orbiting), non-hashed bars indicates contribution of alternative trials (orbiting permitted (fixed-wing), 100 kts airspeed (helicopter)). Contribution of each treatment type has been scaled to account for differences in sample size.

Sighting rates by distance revealed that the fixed-wing observers' most effective sighting range was between 100 and 200 m from their flight path (Fig. 2). Here, they successfully sighted up to 33% of deployed analogues. This rate reduced to 13–14% at distances of 200–300 m from the aircraft. All analogues deployed outside these ranges were sighted less than 9% of the time. Helicopter observers consistently saw a greater proportion of deployed analogues than the fixed-wing observers at distances up to 250 m from the aircraft (Fig. 2). The optimal sighting range for the helicopter was 100 m from the flight path, at which half the deployed analogues were sighted. However at least 25% of analogues were sighted at each distance bin between 50 and 250 m from the flight path. The higher visibility of the helicopter cockpit also enabled observers to sight analogues within 50 m of their flight path. Similar to the fixed-wing observer sightings, distant analogue deployments (more than 250 m from the flight path) were sighted less than 10% of the time (Fig. 2).

Minimal differences were seen in fixed-wing sighting rates for trials where the pilots were permitted to orbit to verify sightings, compared with non-orbiting flights (Fig. 2). Although the predisposition of the observers to expect large shark-like objects in the deployment grid may have contributed to their rapid identification, the sighting cue to perform the verification check (i.e. the decision that a submerged object is likely to be a shark) was sufficient in itself to successfully identify deployed analogues. This suggests there was no ambiguity in the identification of shark analogues during these experiments, nor any disadvantage to the fixed-wing aircrew when not permitting them to orbit suspected sightings as per their normal routine on aerial beach patrols.

Airspeed made minimal difference to the sighting ability of helicopter observers when flying within 250 m of deployed analogues (Fig. 2). However, trials carried out at the faster airspeed (100 kts) resulted in no analogue detections at distances over 300 m from the helicopter flight path. Sighting rates over 300 m were, however, comparable between helicopter observers flying at 60 kts and the fixed-wing observers at 100 kts once sample size was taken into account (Fig. 2). This indicates that the observers from both aircraft types had equivalent, albeit low, capacity to detect distant analogues when traveling at cruising speed.

The sighting of each analogue along each transect run was modelled against all available experimental and environmental variables to determine if such factors influenced analogue sightability. A logistic regression analysis was used against a binary dependent variable (analogue sighted or not). The best fitting and most parsimonious model, based on AIC, incorporated six of the eight variables tested, all of which had a significant effect on analogue sighting (Table 2). Once again the model was tested for heteroscadasticty and normality of the residuals when compared to fitted values. Some care should be taken with the interpretation of the model results however, as the data are unbalanced in that there are far fewer data points for runs where an analogue was actually spotted (Zuur et. al 2009).

**Table 2:** Logistic regression analysis examining the effects of aircraft type, distance to analogue and various environmental variables on analogue sightings per transect.

Coefficients	Estimate	s.e.	z-value	P (>|z|)
(Intercept)	−0.48	0.97	−0.50	0.621
Distance to Analogue	−0.004	0.001	−6.7	<0.001
Sun Position	−0.007	0.001	−6.1	<0.001
Aircraft	3.60	0.56	6.4	<0.001
Wind Speed	0.15	0.06	2.43	<0.05
Cloud Cover	0.58	0.14	3.97	<0.001
Direction (North/South)	−0.40	0.17	−2.36	0.02
Aircraft×Wind Speed	−0.33	0.07	−4.88	<0.001

Residual deviance: 956.5 on 1317 DF, AIC: 974.5.

Nevertheless, the model indicated that distance to the analogue, hour of day (sun position) and whether the observer was facing north (i.e. towards the northerly aspect of the sun) all had a negative impact on whether an analogue was sighted. The model also indicated that sightings were improved by the use of the helicopter compared to the fixed-wing aircraft. The helicopter crew maintain that the slower speeds their aircraft are capable of flying is the primary reason for their improved sighting rate. This suggestion is supported by the lack of distant sighting by helicopter observers at higher speeds (Fig. 2).

The environmental variables of wind speed, cloud cover and turbidity varied considerably during sampling (Table 3). Increased cloud cover aided analogue sightability, as it reduced the effect of sun glare on the water (Table 2). The effect of wind speed is difficult to explain, but is based primarily on its interaction with aircraft type and suggests that the ability of helicopter observers to do a better job of sighting analogues over fixed-wing observers was reduced in higher winds. Turbidity was found to be non-predictive in the distance experiment. However the daily turbidities indicated by the 25 cm secchi disk were invariably greater than the depths at which the 2.5 m shark analogues were deployed (Table 3). It is likely that turbidity would become a significant factor if the analogues were deployed deeper, or if the water was much more turbid. Water depth was found to have no predictive effect on shark analogue sightability (Table 2), supporting our earlier interpretation of this variable's effect in the depth experiment.

**Table 3:** Summary of environmental conditions experienced by each aircraft during horizontal trials.

Aircraft	Day	Wind Speed (kts)	Cloud Cover (%)	Substratum Depth (m)	Secchi Depth (m)
Fixed-wing	1	18.0 (18.0–18.0)	12.5 (12.5–12.5)	11.8 (9.6–13.5)	2.0 (2.0–2.0)
Fixed-wing	2	11.4 (9.5–12.0)	73.4 (50.0–87.5)	12.3 (9.9–15.3)	2.7 (2.5–3.9)
Fixed-wing	3	6.0 (6.0–6.0)	100 (100–100)	12.2 (9.8–14.2)	3.3 (3.1–3.4)
Fixed-wing	4	7.1 (6.3–7.5)	81.0 (62.5–100)	11.9 (9.9–13.9)	3.6 (3.1–4.1)
Fixed-wing	5	5.4 (5.0–5.5)	100 (100–100)	11.7 (9.4–14.6)	4.2 (3.7–5.3)
Fixed-wing	6	9.3 (8.3–10.0)	87.3 (58.4–100)	11.5 (9.3–13.3)	6.3 (5.9–6.6)
Helicopter	1	12.0 (12.0–12.0)	0.0 (0.0–0.0)	12.2 (9.8–14.0)	2.1 (2.0–2.5)
Helicopter	2	7.6 (5.0–10.0)	39.1 (12.5–62.5)	12.0 (9.7–14.9)	2.7 (2.5–3.6)
Helicopter	3	7.0 (5.5–8.0)	97.7 (93.8–100)	12.5 (9.9–14.4)	3.6 (2.8–3.9)
Helicopter	4	7.7 (7.3–8.3)	87.5 (75.0–100)	11.9 (9.8–13.9)	3.7 (3.1–4.2)
Helicopter	5	2.5 (1.5–3.8)	100 (100–100)	11.9 (9.5–14.9)	4.6 (3.9–7.8)
Helicopter	6	11.1 (10.8–11.3)	75.0 (75.0–75.0)	11.8 (9.4–13.6)	6.0 (5.5–9.2)

Values indicate mean for the day, numbers in parenthesis indicate range.

Although we requested observers to identify the ‘species’ of analogues sighted on the last four days of trials, identifications were attempted for only 34% and 55% of sightings for the fixed-wing and helicopter observers, respectively. Importantly, none of the 38 white shark or tiger shark deployments were successfully identified, with only hammerhead shark analogues correctly identified as such. Although the accuracy of identified analogues was relatively high (77% (fixed-wing) and 98% (helicopter)), the low rate of attempts meant that overall, only 26% and 55% of deployments were correctly identified to ‘species’ for the fixed-wing and helicopter observers, respectively.

## Discussion

Aerial beach patrols are often presented as an effective preventative measure against shark attack on humans. They receive considerable public support in Australia, but until now the efficacy of aerial observers in sighting potentially dangerous sharks has not been examined. Undercounting is one of the biggest problems associated with most aerial surveys, with considerable effort taken to convert the numbers of animals sighted to realistic abundance estimates [9], [16], [36]. Although aerial beach patrols are not formal surveys for quantifying the abundances of sharks, as a means for protecting swimmers from attack, they should successfully detect a high proportion of sharks present in the area surveyed. In eastern Australia, operators claim high sighting abilities, as they have “developed and proven and (sic) accurate system for predicting and monitoring the movement of dangerous sharks along our beaches” (www.surfwatchaustralia.com; accessed 25.07.2013). However, the overall sighting rates quantified in the present study of 12.5% and 17.1% for fixed-wing and helicopter observers respectively, suggests that the actual rates of aerial shark sightings fall well short of this claim. Although our study was not conducted along coastal beaches, it was conducted under favourable sighting conditions such as relatively clear, sheltered waters, deployments within 2 m of the surface, flight paths conducted East-West to minimize sun glare, short flight durations to maintain observer attention and the knowledge that shark analogues were expected to be sighted. Observers are unlikely to have such favourable conditions when conducting genuine aerial beach patrols.

Our findings support other studies which have demonstrated the difficulty of detecting marine animals from the air. Marine aerial surveys can potentially miss over two-thirds of surface-oriented fauna, even when animals are travelling in groups [16]. Sighting submerged animals is even more difficult, probably accounting for a high proportion of under-reporting [14], [37]. Marine animals located within sighting distance of aircraft observers can be missed if they are swimming too deep, or are in water conditions which mask their presence. Potentially dangerous species such as white sharks and tiger sharks often orient themselves close to the substratum when inshore [30], [33] and, considering the shallow detection depths found in this study (2.5–2.7 m below the water surface), such sharks may be well inshore of the surf backline before being detectable from the air. Disturbingly, with inshore white sharks preferentially inhabiting water depths shallower than 15 m deep [38], potentially dangerous animals could remain undetectable to aerial beach patrols when in close proximity to surfers and swimmers at many currently-patrolled Australian beaches.

The size of the shark analogues used in this study (2.5 m) were the approximate size at which white sharks change their diet from teleosts to larger prey such as other sharks and mammals [38]. Sharks of this size can be a threat to humans [39], thus our findings estimated the sighting ability of aerial observers for potentially dangerous sharks. We had expected that the greater experience of the fixed-wing aircrew would result in better sighting rates, but this was not the case. Observer experience can influence terrestrial aerial sighting rates by as much as 9% [11], yet no such advantage was apparent for the aircrews in this study. It is possible that an experience effect may have become apparent had we deployed smaller, less-visible analogues, however this was not examined due to the low (<10%) distant sighting rates seen with the full-sized analogues.

Although not tested, it is unlikely that live, moving sharks would be sighted at higher rates, or at deeper depths than our static analogues. Many sharks swim at speeds less than 0.9 ms^−1^ [40]–[42], so their relative movements are negligible compared to that of an aircraft travelling at 100 kts (51 ms^−1^). The difference between these relative movements is magnified the further the shark is from the aircraft's flight path. Similarly, the body movement of a live shark is unlikely to contribute greatly to its sightability. A beating tail represents only a small proportion of a shark's body area, and species such as the tiger shark may beat their tail less than once every two seconds, or occasionally undertake “powerless” glides, with the tail held stationary [40].

The risk of shark attack is thought to be heightened during conditions of low visibility, such as at dawn and dusk. During these times, the low angle of the sun can increase sun glare, which can significantly reduce marine aerial sightings [19]. Observers in our study reported sun glare sometimes being problematic in this study, a result that was supported by our finding that transects flown with the observers facing north (towards the sun) had reduced numbers of analogue sightings. Low sun angle also limits light penetration into the water, which is likely to further reduce the visibility of sub-surface sharks to aerial observers. High wind speeds affected helicopter sightings more than fixed-wing sightings, although the helicopter observers still saw a greater proportion of deployed analogues.

The results of the modelling indicated that sighting rates were affected by environmental conditions such as sun glare, cloud cover and wind speed. Yet the dependence on favourable weather for aerial patrols can also severely restrict their efficacy by reducing the number of days flown. Inclement environmental conditions are unlikely to reduce predatory shark behaviours, although it may reduce the number of beach users. If, however, environmental conditions such as fog or high winds dissipate during the course of a day, perfect beach weather may result in greater numbers of swimmers, yet aerial observers may not have flown.

In addition to having demonstrated limited sighting rates, aerial beach patrols also survey individual beaches for very short periods of time each day. For example, the current NSW fixed-wing aerial patrols survey long distances (∼285 km) on their regular flights (www.aerialpatrol.com.au). This, plus their average cruising speed of 100 kts, means that the aircraft are above any single beach for only minutes each day. Although the public may feel safer knowing that aircraft are in the air, the tangible difference these flights make to an individual bather's safety from shark attack at any one of these beaches is likely to be small. While coastal aerial patrols can provide benefits in terms of other surveillance, such as locating vessels or fishers in distress, monitoring marine pollution and assisting in beach rescue operations, these benefits remain secondary to the primary task of shark detection.

Clearly, alternative safety measures to aerial beach patrols should be considered to protect the public from shark attacks. These may still include alternative aerial systems; Overhead video observation systems (‘blimp-cams’) have the capacity to determine marine activities for up to 200 m around unmanned platforms [43]. These systems use remotely-operated cameras to stream live signals to observers, and have been used to successfully investigate the movements of marine mammals such as dugongs [44]. Although blimp-cams have limited mobility, such systems could mean continuous monitoring of a section of beach. While historically used for military applications, a recent expansion of civilian-oriented unmanned aerial vehicles (UAVs) also provides potential platforms which could supplement current airborne operations. The application of such methods to detect inshore sharks has yet to be determined, however they are worth further investigation.

The utility of lifeguards in sighting sharks has also been demonstrated with sharks identified up to 300 m offshore by lifeguards atop 3 m beach towers around Oahu, Hawaii [45]. Similarly, a very successful shark detection program has been developed in Cape Town, South Africa, using trained observers on vertically-elevated structures such as surrounding cliffs and buildings to alert the public when a potentially dangerous shark is seen approaching beaches [46]. Programs such as these are most effective at sloping beaches with extended regions of shallow waters, allowing sharks to be detected at greater distances from the shore. With nearly 40 coastal drownings per year in NSW, compared to only two coastal shark fatalities over 35 years [47], [48], it is apparent that sharks pose significantly less threat to human life than other beach-related activities. Enhancing existing lifesaving programs may therefore ultimately do more to reduce the overall number of fatalities at beaches as well as assisting with other medical emergencies. Further research into the movements and behaviours of sharks is also a logical step to reduce future problematic interactions between sharks and humans.

In conclusion, we found severe limitations in the ability of aerial observers in fixed-wing aeroplanes and helicopters to sight shark analogues, both in terms of depth and distance from the aircraft, and when environmental conditions were not ideal. We gave our aircrews the best opportunities to sight shark analogues (deploying large analogues at shallow depths, with observers alert and aware of their presence within a small area), yet even with such optimal conditions, relatively few analogues were sighted. Although it is acknowledged that aerial patrols do detect coastal sharks on occasion [49], the low rates of detections found in this study cast serious doubts on their use as an effective early-warning detection system. The results from our experiments suggest that aerial patrols provide limited realistic benefit in terms of bather safety, while giving the public an inflated sense of protection against shark attacks.
